# Biofilms and exopolysaccharides in *Pseudomonas aeruginosa*: pathogenesis, immune evasion, and lung–brain signaling during pneumonia

**DOI:** 10.1038/s41392-024-01902-4

**Published:** 2024-08-19

**Authors:** Shuaibing Zhang, Pierre Stallforth

**Affiliations:** 1 Department of Paleobiotechnology, Leibniz Institute for Natural Product Research and Infection Biology – Hans Knöll Institute, Beutenbergstraße 11a, D-07745 Jena, Germany; 2 Institute for Organic Chemistry and Macromolecular Chemistry, Friedrich Schiller University Jena, Humboldtstraße 10, D-07743 Jena, Germany; 3 Cluster of Excellence Balance of the Microverse, Friedrich Schiller University Jena, Fürstengraben 1, D-07743 Jena, Germany

**Keywords:** Infectious diseases, Infection

A recent study by Granton et al. in *Cell* investigates the role of exopolysaccharide (EPS) in *Pseudomonas aeruginosa*’*s* biofilm formation, immune evasion, and lung–brain signaling during pneumonia.^1^ This research significantly advances our understanding of how *P. aeruginosa’s* biofilms influence disease symptoms and suggests potential therapeutic strategies targeting the lung–brain axis to improve clinical outcomes in pneumonia patients.

Long before the COVID-19 pandemic, social distancing from individuals suffering from severe respiratory tract infections has been an effective strategy to limit the transmission of airborne diseases.^2^ In their work, Granton et al. investigate the specific mechanisms by which the bacterium *P. aeruginosa* triggers symptoms of sickness in individuals suffering from pneumonia. Understanding these mechanisms is vital as they directly influence the spread of the disease. Bacterial strains causing less noticeable sickness may result in symptoms undetected by others, eventually enhancing the transmission of the pathogen.

*P. aeruginosa* is a ubiquitous and highly adaptable bacterium, which can thrive in a huge variety of environments. It is capable of causing a range of infections in humans, particularly of the respiratory tract of individuals suffering from cystic fibrosis.^3^ Much of its ability to survive in diverse habitats is due to the bacterium’s ability to form strong biofilms, enabled by the production of two key exopolysaccharides: Pel (pellicle) and Psl (polysaccharide synthesis locus).

In their study, the authors engineered two near isogenic strains of *P. aeruginosa* PAO1; one producing Pel and Psl at 37 °C (referred to as EPS^+^) and one unable to generate these components at any temperature (referred to as EPS^−^). The EPS^+^ strain formed biofilm-like aggregates at 37 °C and remained unaggregated at 25 °C. This temperature-induced production of EPS allowed the authors to handle the strain at 25 °C as planktonic cells thus facilitating precise inoculations. The authors also used a virulent *Escherichia coli* strain as a control pathogen.

When infected with these two *Pseudomonas* strains, mice showed different responses. Those animals subjected to the EPS^−^ strain developed severe pneumonia with pronounced sickness, hypothermia, and behavioral changes. Interestingly, the study noted sex-dependent differences in sickness response of mice infected with EPS^−^. The research team, however, found no direct correlation between disease manifestation and specific soluble inflammatory mediators.

The study highlighted that the EPS layer of the bacterium effectively coats the lipopolysaccharide (LPS) layer and hides it from immune recognition. LPS is a key virulence factor recognized by cells *via* the Toll-like receptor TLR4. In agreement with this hypothesis, TLR4^–/–^ mice showed indeed a different response to LPS-exposed EPS^−^ bacteria compared to the EPS^+^ strain with ‘hidden’ LPS. Signs of sickness were decreased in TLR4^–/–^ mice infected with EPS^–^ compared to wild type mice. For EPS^+^ infections little difference was observed between wild type and TLR4^–/–^ mice.

A very important finding of this study was the identification of a signal transduction pathway from the lung to the brain mediated by TRPV1^+^ sensory neurons expressing TLR4. These neurons effectively link the LPS-TLR4 response to sickness. The researchers demonstrated that the actual cause of sickness is not inflammation itself but rather the activation of stress responses in the paraventricular nuclei of the hypothalamus. This activation of corticotropin-releasing hormone neurons ultimately drives the sickness response, emphasizing the complex interplay between microbial infection, immune response, and neurological signaling in disease manifestation. This finding has profound implications for understanding the pathophysiology of infectious diseases and for pioneering novel therapeutic strategies targeting the lung–brain axis. Specifically, interventions aimed at inhibiting microbial detection by sensory nerves, thereby reducing the activation of stress pathways, demonstrate potential in mitigating illness severity and improving clinical outcomes in severe cases of pneumonia.

**Fig. 1:**
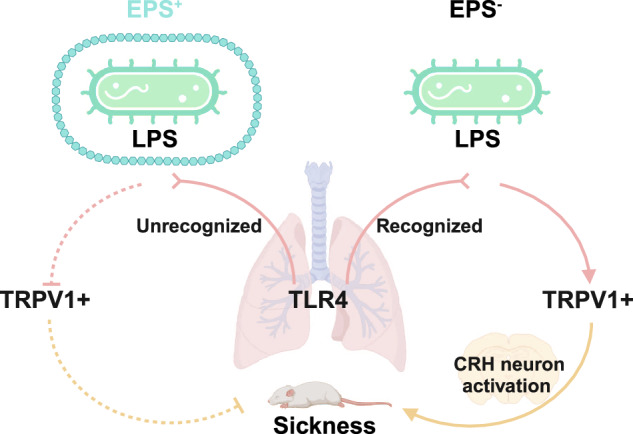
EPS-negative *Pseudomonas aeruginosa* strains are implicated in severe illness by activating lung sensory neurons through exposed lipopolysaccharide (LPS), which binds to Toll-like receptor 4 (TLR4) on TRPV1^+^ sensory neurons. This activation initiates acute stress responses in the brain, mediated by CRH neurons, leading to sickness behavior. In contrast, EPS-producing biofilm pathogens evade this sensory response, facilitating their persistence with the host environment. The figure is created with BioRender.com

In summary, Granton et al. reveal a complex interplay between *P. aeruginosa* biofilms, EPS production, and host immune response (Fig. 1). While infections with EPS^-^ cells, which do not form biofilms, are perceived as more severe, biofilm formation can also lead to tissue destruction^4^ and, crucially, allows for the persistence of the pathogen. As these bacteria are effectively concealed from sensory neurons, infected individuals may display only mild signs of sickness. The research provides a significant advancement in our understanding how the complex lifestyle of *P. aeruginosa* affects pneumonia symptoms in patients infected with this pathogen, which may give us some instructions for the development of effective therapeutic strategies, for instance, by targeting the ability of biofilms to evade host detection and modulate host responses, we may able to develop new treatments that improve clinical outcomes in patients with these infections. Furthermore, future studies could examine the signaling pathway in cystic fibrosis (CF)-relevant models, such as β-ENaC transgenic mice, to simulate CF lung disease. Utilizing clinical isolates from CF patients can further highlight the relevance of the findings, potentially leading to better treatments for CF lung infections.^5^ Additionally, their study poses interesting questions that remain unanswered, such as why neutrophils are essential for survival of EPS^+^ *P. aeruginosa* and the mechanisms behind sex-based differences in the response of mice infected with *P. aeruginosa*.
